# Search for an Endogenous Bombesin-Like Receptor 3 (BRS-3) Ligand Using Parabiotic Mice

**DOI:** 10.1371/journal.pone.0142637

**Published:** 2015-11-12

**Authors:** Dalya M. Lateef, Cuiying Xiao, Marc L. Reitman

**Affiliations:** Diabetes, Endocrinology, and Obesity Branch, National Institute of Diabetes and Digestive and Kidney Diseases, National Institutes of Health, Bethesda, Maryland, 20892, United States of America; University of California, San Francisco, UNITED STATES

## Abstract

Bombesin-like receptor 3 (BRS-3) is an X-linked G protein-coupled receptor involved in the regulation of energy homeostasis. *Brs3* null (*Brs3* ^*-/y*^) mice become obese. To date, no high affinity endogenous ligand has been identified. In an effort to detect a circulating endogenous BRS-3 ligand, we generated parabiotic pairs of mice between *Brs3* ^*-/y*^ and wild type (WT) mice or between WT controls. Successful parabiosis was demonstrated by circulatory dye exchange. The *Brs3* ^*-/y*^-WT and WT-WT pairs lost similar weight immediately after surgery. After 9 weeks on a high fat diet, the *Brs3* ^*-/y*^-WT pairs weighed more than the WT-WT pairs. Within the *Brs3* ^*-/y*^-WT pairs, the *Brs3* ^*-/y*^ mice had greater adiposity than the WT mice, but comparable lean and liver weights. Compared to WT mice in WT-WT pairs, *Brs3* ^*-/y*^ and WT mice in *Brs3* ^*-/y*^-WT pairs each had greater lean mass, and the *Brs3* ^*-/y*^ mice also had greater adiposity. These results contrast to those reported for parabiotic pairs of leptin receptor null (*Lepr* ^*db/db*^) and WT mice, where high leptin levels in the *Lepr* ^*db/db*^ mice cause the WT parabiotic partners to lose weight. Our data demonstrate that a circulating endogenous BRS-3 ligand, if present, is not sufficient to reduce adiposity in parabiotic partners of *Brs3* ^*-/y*^ mice.

## Introduction

Bombesin-like receptor 3 (BRS-3) is an X-linked G protein-coupled receptor that is located chiefly in certain brain regions, including those regulating food intake and metabolic rate [1–6]. Genetic and pharmacologic studies have demonstrated a role for BRS-3 in the regulation of energy metabolism, body temperature, insulin secretion, blood pressure, and heart rate [1,3,7]. For example, *Brs3* null (*Brs3* ^*-/y*^) mice have a reduced fasting metabolic rate, resting heart rate, and body temperature, and increased food intake and obesity [3,7–11]. Conversely, synthetic selective BRS-3 agonists increase fasting metabolic rate, blood pressure, and heart rate and reduce food intake and body weight in mice [3,12]. The agonists require continuous high-level receptor occupancy for weight loss efficacy, indicating that tachyphylaxis does not occur [3,12].

BRS-3 has a low affinity for bombesin and is one of hundreds of mammalian GPCRs (most are olfactory GPCRs) that do not have an identified endogenous ligand [13]. The mammalian GPCRs most closely related to BRS-3 bind neuromedin B and gastrin-releasing peptide with nanomolar affinity, but these ligands bind BRS-3 with 1000-fold lower affinity [14]. CCHamide-1 and CCHamide-2 are highly potent peptide agonists for two insect BRS-3 homologues [15,16]. While it seems plausible that mammalian BRS-3 has a peptide ligand, no high-affinity endogenous ligand for BRS-3 has been identified to date. Attempts to deorphanize mammalian BRS-3 [13] have identified only relatively low affinity agonists, including hemorphins [17] and peptide E [18].

Given the lack of success in deorphanizing mammalian BRS-3, we undertook a complementary approach, using parabiosis, the surgical union of two animals to produce circulatory exchange. Parabiosis allows a factor released from one mouse to exert its effect in the partner, thereby indicating the existence of a sufficiently stable circulating endogenous factor [19–21]. Ablation of a receptor often increases the level of its cognate ligand. Parabiotic mice were instrumental in the discovery of leptin, providing the entry point for most of our current understanding of the physiology of obesity [22,23]. Indeed, leptin treatment of one member of a parabiotic pair has an amplified effect on the partner, as compared to treatment of a single mouse [24]. We have produced parabiotic mice in an attempt to detect an endogenous BRS-3 ligand.

## Materials and Methods

### Mice and study design

Male C57BL/6 mice were purchased from The Jackson Laboratory (Bar Harbor, ME), and *Brs3* ^*-/y*^ mice were provided by Dr. James Battey [9] and back-crossed at least eight generations onto a C57BL/6J background. Mice were housed at 21–22°C in a temperature- and humidity-controlled environment with a 12:12-h light-dark cycle, with ad libitum access to food and water. Before surgery, mice were maintained on chow (7022 NIH-07 diet, Harlan laboratories) and after surgery, on a high fat diet (D12492, 60% kcal fat; Research Diets, New Brunswick, NJ). All animal studies were approved by the National Institute of Diabetes and Digestive and Kidney Diseases Institutional Animal Care and Use Committee.

### Parabiosis

Weight-matched male mice at 6–10 weeks of age underwent surgery to make parabiotic pairs [25]. Briefly, under ketamine/xylazine anesthesia and after clipping hair and prepping with betadine, a lateral longitudinal incision was made along opposing sides of two mice. Skin was freed and femora were exposed by blunt dissection, avoiding damage to muscle and nerves. Periosteum was scraped off half the length of each femur and the bones were pulled together by suturing twice around them using 2–0 silk. Muscles around the bones were joined with three deep sutures. The scapulae were exposed, periosteum scraped, and the scapulae were joined by suturing through the bones three times using 4–0 silk. Post operatively, mice were given banamine (2.2 mg/kg s.c.) analgesia daily for three days. Of the 18 pairs created, one died due to accidental anesthesia overdose and two were excluded as technical failures (due to lack of fusion of the scapulae, ascertained by observation at 4 weeks after surgery). Pairs were housed singly with food on the floor of the cage.

### Parabiosis verification

Circulatory exchange between parabiotic pairs was measured by dye exchange 7 weeks post-operatively [26]. Briefly, 400 μl of 0.5% Evans blue dye in Hanks’ Salt Solution was injected into the tail vein of one mouse and blood was collected from tail veins of both mice 30 and 60 minutes later. Exchange was calculated by numerical solution of the equation Coth rt = Ci/Cr, where Coth is the hyperbolic cotangent, r is rate of exchange expressed as the fraction of one animal’s plasma volume per minute, t is the time since injection, and Ci and Cr are the A_620_ (serum absorbance at 620 nm) of injected and recipient mice, respectively.

### Body composition

Body composition was measured by X-ray absorptiometry (Lunar PIXImus with Lunar PIXImus2 version 2.1 software, GE, Madison, WI). Anesthetized (ketamine/ xylazine) parabiotic pairs were imaged twice in the prone position, with repositioning between the scans and averaging the two scans. Reproducibility was similar for mice as individuals vs in a pair.

### Post mortem analysis

Ad lib fed pairs were anesthetized (ketamine/xylazine) 3–4 hours into the light period and blood was collected retroorbitally. After cervical dislocation, the mice were separated and lean and fat mass were measured by X-ray absorptiometry. The gastrointestinal tract was weighed, emptied, and reweighed as an index of food intake [25].

#### Statistics

Results are shown as mean ± SEM. Two-way ANOVA with or without repeated measures followed by Holm-Šídák’s posttest. Student’s t-test was used when two groups were compared. Statistical analyses used two-tailed tests using *P* < 0.05 as statistically significance.

## Results

**Fig 1:**
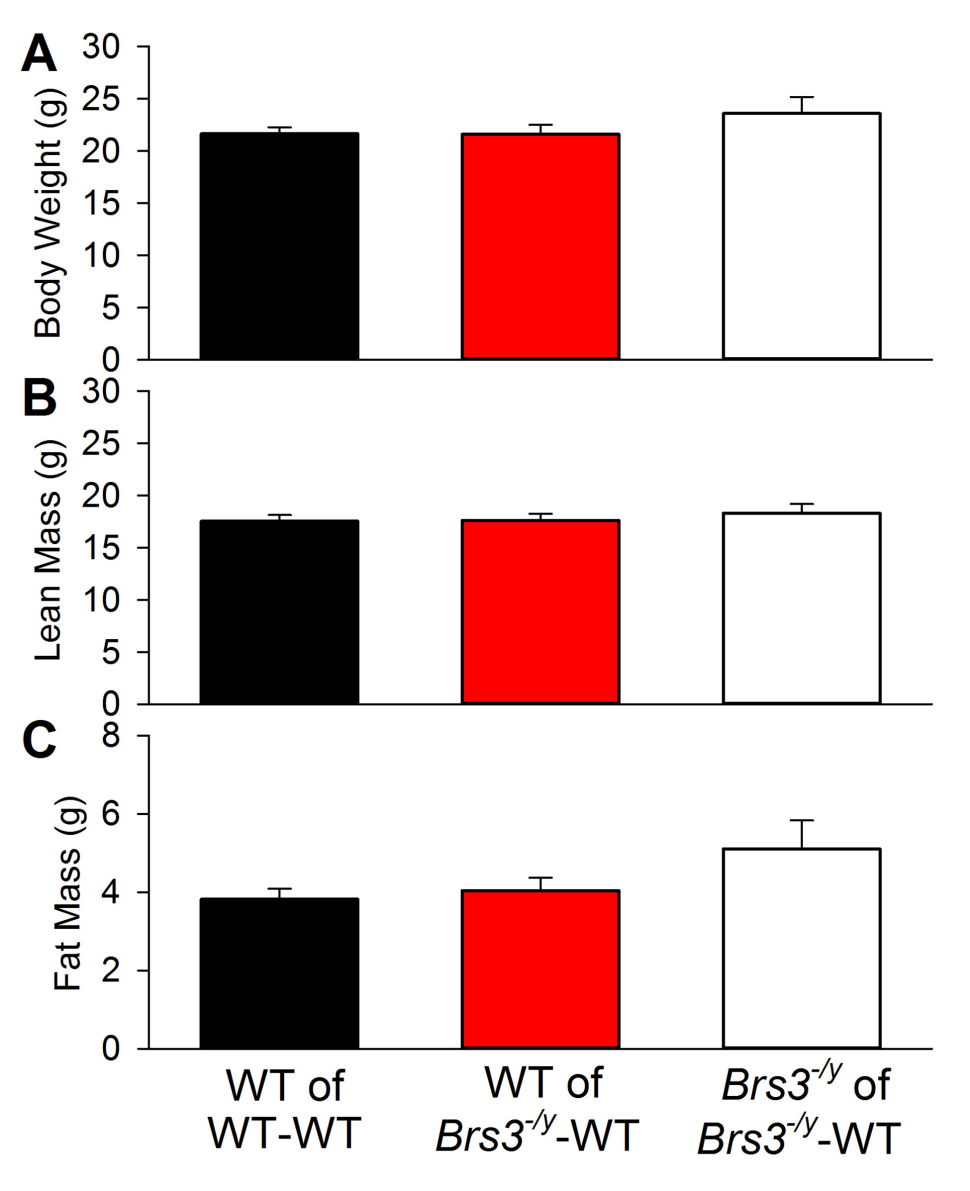
Initial body weight and composition of WT and *Brs3* ^*-/y*^ mice. Body weight (A), lean mass (B), and fat mass (C) of the indicated mice at the time of parabiosis surgery. Data are mean ± SEM; N = 7-14/group. There are no significant differences between the groups.

**Fig 2:**
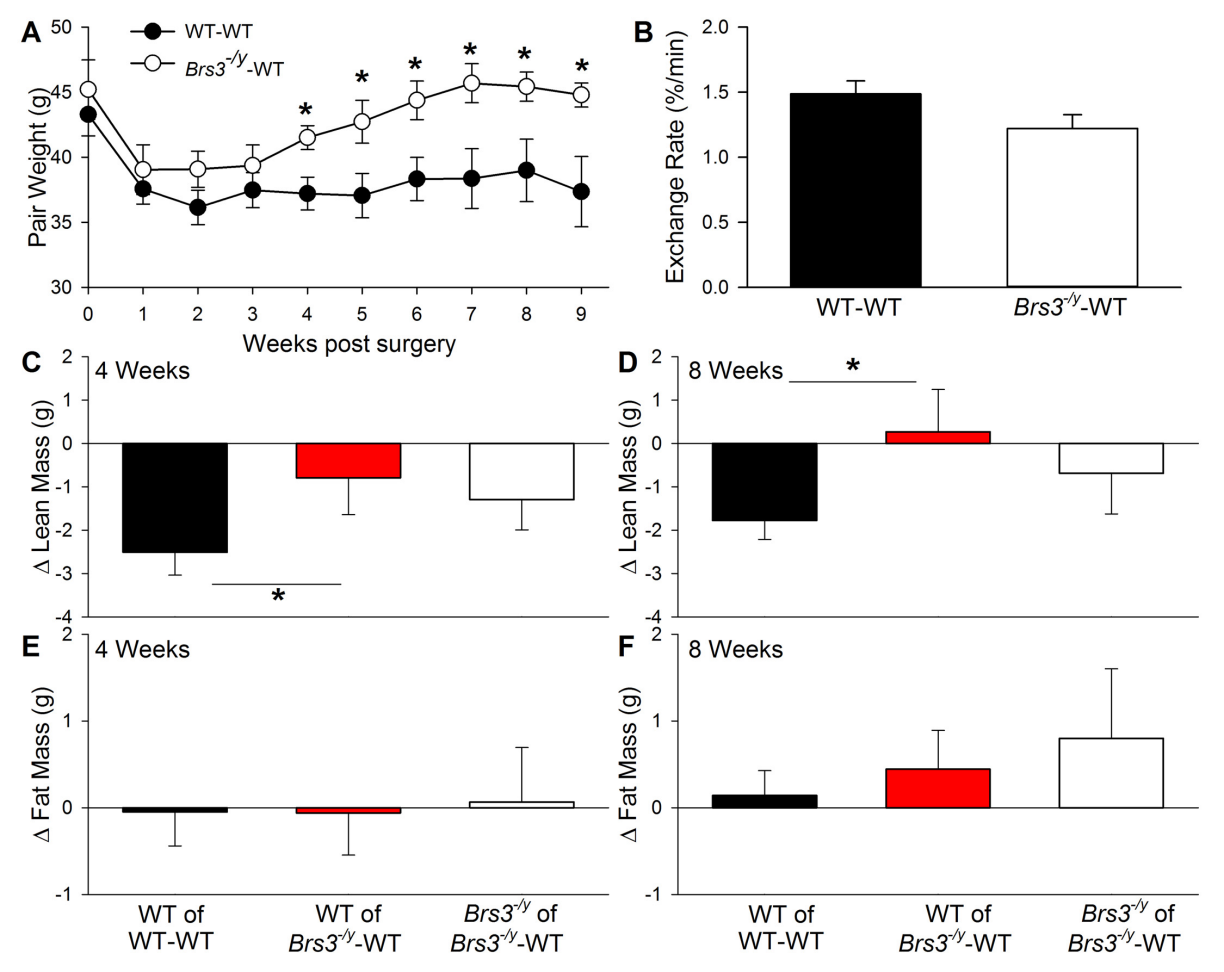
Body weight and composition in parabiotic pairs. (A) Pair weights after parabiosis. (B) Circulatory exchange between parabiotic mice at 7 weeks after surgery, determined by Evans blue transfer. Change after parabiosis in (C, D) lean mass and (E, F) fat mass at 4 or 8 weeks after surgery. Data are mean ± SEM; N = 7–8 pairs/group. * *P*<0.05.

A wild type (WT) mouse was surgically joined with either another WT mouse creating WT-WT parabiotic pairs or with a *Brs3* ^*-/y*^ mouse creating *Brs3* ^*-/y*^-WT pairs. At the time of surgery, mice were 6–10 weeks old, with similar body weight, lean mass, and fat mass (Fig 1). By the day after surgery, mice were active and, although joined with another mouse, showed locomotive behavior that appeared comparable to that of single mice. All pairs lost weight in the week after surgery, after which WT-WT pairs stabilized while the *Brs3* ^*-/y*^-WT pairs gained some weight (Fig 2A).

Circulatory exchange between parabiotic mice was 1.32 ± 0.10% of plasma volume/min (range 0.77–2.15%) and was comparable in WT-WT and *Brs3* ^*-/y*^-WT pairs (Fig 2B). This exchange rate is similar to previous reports [21], demonstrating that parabiosis was achieved successfully.

Body composition was measured using X-ray absorptiometry, allowing evaluation of individual mice in parabiotic pairs. Lean mass of WT mice of WT-WT pairs was lower than before surgery (-2.5 ± 0.5 g at 4 weeks and -1.8 ± 0.4 g at 8 weeks). There was less reduction in lean mass in WT mice in *Brs3* ^*-/y*^-WT pairs (-0.8 ± 0.8g at 4 weeks and +0.3 ± 1.0g at 8 weeks, both P<0.05 vs WT in WT-WT) (Fig 2C and 2D). Fat mass was not significantly different between any of the groups at either time point (Fig 2E and 2F).

**Fig 3:**
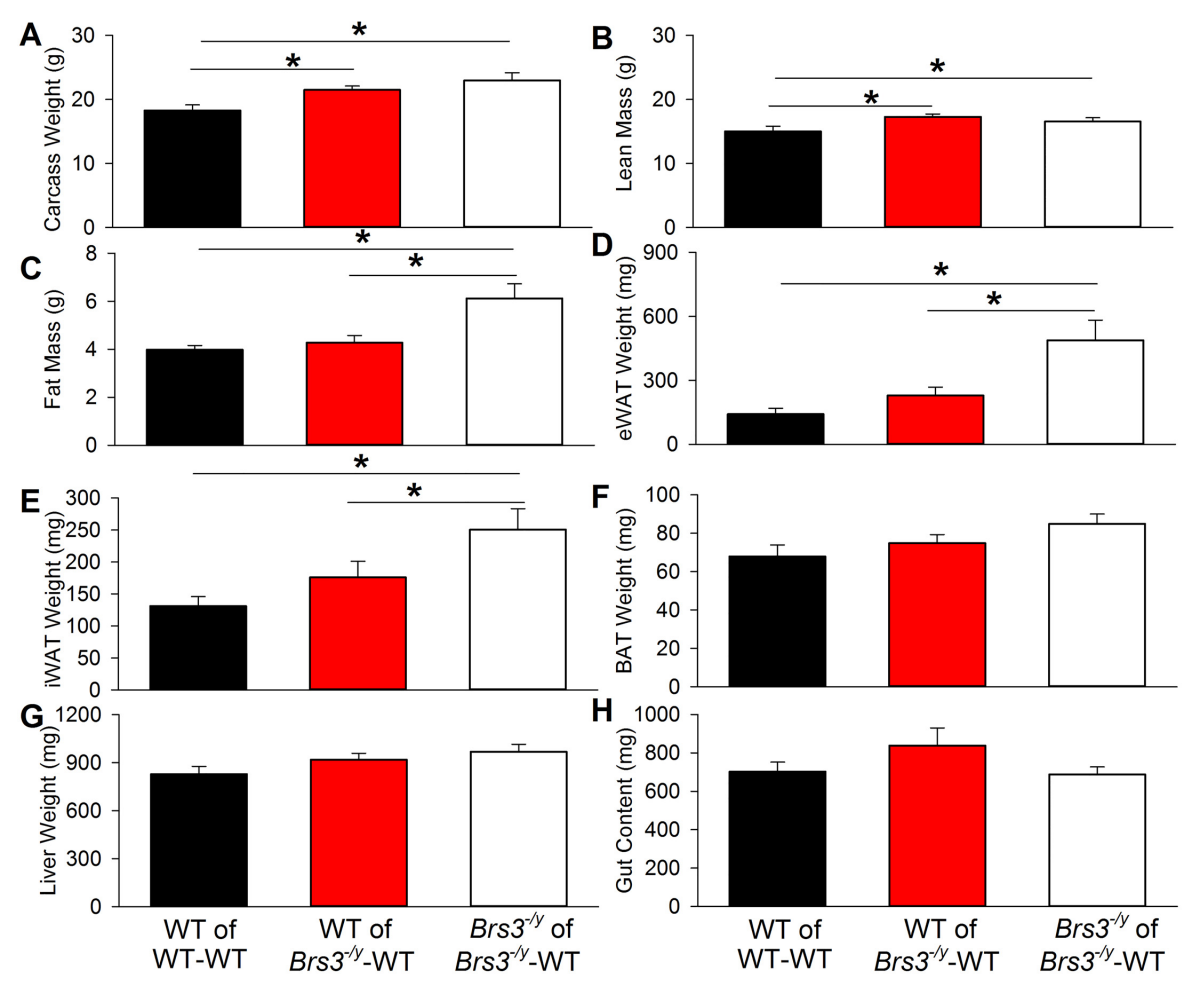
Effects of parabiosis at 9 weeks. (A) Body weight, (B) lean mass, (C) fat mass, (D) epididymal white adipose tissue (eWAT) weight, (E) inguinal white adipose tissue (iWAT) weight, (F) brown adipose tissue (BAT) weight, (G) liver weight, and (H) gut content. Data are mean ± SEM; N = 8–14 mice/group. * *P*<0.05.

The parabiotic pairs were euthanized at 9 weeks after surgery. Both the carcass weight and lean mass of WT mice in WT-WT pairs were less than in either WT or *Brs3* ^*-/y*^ mice in *Brs3* ^*-/y*^-WT pairs (Fig 3A and 3B). Fat, measured as total, epididymal, or inguinal, was significantly greater in *Brs3* ^*-/y*^ mice in *Brs3* ^*-/y*^-WT pairs than in WT mice in either WT-WT or *Brs3* ^*-/y*^-WT pairs (Fig 3C–3E). There was no difference in brown adipose tissue weight, liver weight, or gut content weight between any of the groups (Fig 3F–3H).

## Discussion

We tested for the presence of an endogenous BRS-3 ligand using parabiotic pairs of mice. Exogenous treatment with synthetic BRS-3 agonists causes a reduced food intake and body weight [3,12]. We hypothesized that *Brs3* ^*-/y*^ mice might have increased circulating ligand concentration similarly to mice with ablation of other receptors, and that WT mice, when joined to a *Brs3* ^*-/y*^ mouse, might eat less and have lower adiposity and body weight due to endogenous BRS-3 ligand circulating from the *Brs3* ^*-/y*^ mouse. However, the WT partner in the *Brs3* ^*-/y*^-WT pairs did not lose weight, and actually had a higher lean weight and similar (but not lower) adiposity compared to control WT mice in WT-WT pairs. The higher lean mass of WT mice in *Brs3* ^*-/y*^-WT, compared to WT-WT, pairs, may be attributable to better nutrient status of the *Brs3* ^*-/y*^ mouse being passed to its WT partner (although this is not a major effect in the WT mice in *Lepr* ^*db/db*^-WT or *Lep* ^*ob/ob*^-WT parabiotic pairs [20,21]). It is also possible that longer or more frequent visits to food by the *Brs3* ^*-/y*^ member could encourage eating in its parabiotic partner. In summary, circulating endogenous BRS-3 ligand, if present, is not sufficient to reduce adiposity in parabiotic partners of *Brs3* ^*-/y*^ mice.

The failure to detect an existing endogenous BRS-3 ligand in the parabiotic mice could be due to limited cross-circulation and a ligand’s short half-life. With successful parabiosis the inter-mouse circulatory exchange is only 1 to 2% per minute [21], which is ~0.2% of cardiac output [27]. Tachyphylaxis to continuous ligand could be another explanation, but seems unlikely due to the body weight efficacy of continuous agonist dosing experiments [3,12]. Also plausible is that endogenous ligand may not reach the circulation, but rather function as a paracrine factor or neurotransmitter. Most of the BRS-3 in the brain is located in regions behind the blood-brain barrier, consistent with a neurotransmitter paradigm [1–6], however BRS-3 is also located outside the brain [7,14], including pancreatic islets [28]. Alternatively, an endogenous ligand may not exist, with BRS-3 constitutive activity [29] functioning to increase signaling tone. While our data do not provide evidence for an endogenous ligand, the evolutionary conservation of BRS-3 ligand binding activity and specificity for synthetic ligands strongly indicate that there is an endogenous mammalian ligand, somewhere, waiting to be found.

## Abbreviations

WT: wild type
eWAT: epididymal white adipose tissue
iWAT: inguinal white adipose tissue
